# An Evaluation of the Usefulness of YouTube® Videos on Crown Preparation

**DOI:** 10.1155/2022/1897705

**Published:** 2022-01-22

**Authors:** Syed Rashid Habib, Aleshba Saba Khan, Mohsin Ali, Essam Abdulla Abutheraa, Ahmad khaled alkhrayef, Faisal Jibrin Aljibrin, Nawaf Saad Almutairi

**Affiliations:** ^1^Department of Prosthetic Dental Sciences, College of Dentistry, King Saud University, Riyadh, Saudi Arabia; ^2^Department of Prosthodontics, Shahida Islam Dental College, Lodhran, Pakistan; ^3^Department of Comprehensive Dentistry, Case Western Reserve University School of Dental Medicine, Cleveland, Ohio, USA; ^4^College of Dentistry, King Saud University, Riyadh, Saudi Arabia

## Abstract

**Background:**

You Tube is one of the most commonly used online sources for sharing information and knowledge. Academic topics or clinical data shared on this platform is not peer reviewed or evaluated by subject specialists for accuracy. No study was found in the literature examining the validity of crown preparation videos available at this platform.

**Objective:**

To evaluate the authenticity of the content and quality of the crown preparation videos uploaded on the YouTube.

**Methods:**

The systematic search for YouTube videos was carried out over a period of one year from January 2020 until February 2021. The keywords or phrases and tags used were crown preparation, PFM crown preparation, all ceramic crown preparation, and dental crown preparation. The videos were shortlisted on the basis of inclusion and exclusion criteria to select educationally useful videos in terms of content and quality.

**Results:**

Three subject specialists evaluated the videos on crown preparation three times to shortlist only 12 (11%) educationally useful videos out of 109 relevant videos. These 12 videos met the preset inclusion criteria.

**Conclusion:**

Although YouTube is the most popular social media platform used as the source of information by the students, the majority of uploaded content lacks authenticity. This study found that crown preparation videos uploaded by the faculty members or subject specialists can be considered as the reliable source.

## 1. Introduction

The undergraduate dental education, as with any other educational program, needs to adapt evolving techniques and materials in the curriculum in order to fulfill the changing needs of the dental practice [1]. Albeit the intricacies of the dental care have increased significantly over the last century, the strategy of teaching medicine has barely changed [2]. Recently, there is a universal interest in the assessment of the learning strategy since its adoption [2, 3].

The predoctoral dental syllabus generally targets the development of psychomotor dexterity of students in their initial clinical years [3]. This approach is particularly pertinent in prosthodontics in which preclinical practical expertise plays a significant role [4]. Students are therefore, exposed to the technical part of the skills to improve the understanding of clinical procedures [3, 4]. Courses which focus on the improvement of dental laboratory skills are commonly studied in the preclinical years, as these assist in the preparation of students for clinical prosthetic dentistry [2, 4]. According to Alqahtani et al., an in-person illustration to a little gathering has been proven to be helpful in teaching dental laboratory expertise [5]. It enhances courage and assurance in the student, boosts conversation abilities, and contributes to deeper knowledge and better learning of procedures as compared to didactic training [5, 6]. Nonetheless, the same authors have reported that the live demonstration teaching strategy has multiple disadvantages like student's dependence on the teacher, problem in visualization of the technique, and slight fluctuation of the technique between various trainers [5, 6]. Furthermore, the efficacy of demonstrations relies on the number of students supervised by each teacher and the measure of time spent on the demonstration [5]. In addition, though the demonstrations of the procedures are done only once, some students might require multiple demonstrations to learn the fundamental skills [5]. One study also concluded that the conventional educational techniques lead to psychological stress, which might have an impact on severity of nervous exhaustion, anxiety, and distress among students [6].

The preference of video-assisted clinical instruction in dentistry (VACID) over conventional education for learning practical skills has been reported in some studies [7, 8]. Akhlaghi et al. utilized video recordings to educate the patients and students and reported that the students have shown more acceptance towards this strategy which is likely ascribed to the better observation of clinical techniques and enhanced understanding of details [7]. They concluded that VACID has shown better outcomes as compared to traditional methods for education in dentistry [7, 8].

Towards the end of 2019, the coronavirus disease surfaced as an acute respiratory infectious disease in China which was later declared by the World Health Organization (WHO) as a pandemic with high fatality and morbidity rates [9, 10]. The prevailing conditions lead to the popularity in idea of e-learning in dentistry [11]. e-Learning incorporates an assortment of different modalities and terms like learning through internet, mobile education, computer-based learning, distant learning, e-instructing, arbitrate learning, and reflective and virtual learning [8, 11]. With present day innovation, students can easily reach out to the content of lectures while staying at home. This does not require the physical attendance at institutes, thus reducing the chances of transmission of infection [8, 12]. In certain forms, internet-based education aids in self-learning among students and further modifies their approach for knowledge acquisition [8, 13, 14].

During these situations, YouTube audio-visual recordings became convenient and an accessible alternative to the study materials [8, 15] and more acceptable as a teaching strategy [11, 15]. YouTube videos can be found on various topics and by variable sources. This study assumed that there are potential variances concerning the quality of available videos of complete crown preparation. The authors were looking for crown preparation videos with good visualization of the preparation steps along with sound educational narration and content as per universally accepted standard text books [16, 17].

Therefore, the aim of this study was to evaluate the quality and reliability of the information provided in the crown preparation videos currently available on YouTube. No study was found in the literature that has reported the efficacy of YouTube videos on the topic of crown preparation.

## 2. Material and Methods

### 2.1. Criteria for Identification of YouTube Videos and Importance of Selection

The research was conducted as a cross-sectional analysis. The systematic search for YouTube videos was carried out over a period of one year from January 2020 until February 2021. The keywords or phrases and tags used were crown preparation, PFM crown preparation, all ceramic crown preparation, and dental crown preparation.

### 2.2. Data Collection and Assessment of Quality of YouTube Videos

The following data was collected about the videos: name of the uploader and publisher of the video, video title, YouTube video links, total number of the views of the video, total number of likes or dislikes of a video, total number of positive and negative comments (if any) related to the video, date of uploading, total length of the video, video quality, sound quality, the prepared tooth by using the FDI notation system, type of preparation, the type of prepared tooth, qualification of the presenter, primary or permanent tooth, the language used by the presenter, and the quality of English language. For data collection, we explored “http://www.youtube.com” using the google chrome web browser. Inclusion and exclusion criteria helped in shortlisting only 12 videos out of 109 available videos.

### 2.3. Inclusion/Exclusion Criteria (Figure 1)

All videos that fulfilled the predetermined criteria were included. Content must be relevant and scientifically valid reflecting an acceptable knowledge about crown preparation. The videos must include crown preparation (animation or actual preparation on extracted teeth or in clinic on the patient) as well as presented (if there is a presenter) in comprehensible English language. The videos presented by the faculty members and on tooth preparation done on permanent teeth (natural or ivorine) were included. The authors agreed to include videos that followed the ideal crown preparation parameters. The contents regarding crown preparations to be present in the videos were expected to be based on standard guidelines as described in standard text books for fixed prosthodontics and recently reported in the literature [16–18]. The amount of reduction for all ceramic crown preparations was expected to be the following: axial reduction (1.5 mm), occlusal/incisal reduction (2 mm), functional cusp bevel (posterior teeth), marginal design (deep chamfer/radial shoulder), taper between axial/proximal walls (6°), and smooth finish and rounded line angles.

British as well as American accent, both were considered acceptable for inclusion in the study. Minor variations were ignored. If the video was in the English language (and not any other language, i.e., Chinese, Arabic, French, etc.) and the purpose was clearly understood, it was included in the study. The main factor to be assessed was the comprehensible English language. Senior faculty members who have been involved in teaching the subject in English for five or more years were involved in the assessment of videos.

Out of the available videos, poor quality videos (in terms of sound or picture quality below 480 pixels) and non-English language videos were excluded. Among the videos with exactly the same or duplicated content, the videos with lower views were excluded. Videos with more than 5 negative comments were excluded. Other videos in the exclusion criteria were the ones providing information without scientific justification. Two of the most commonly recommended books for fixed prosthodontics, *Contemporary Fixed Prosthodontics* and *Fundamentals of Fixed Prosthodontics*, were kept as the standard [17, 18].

## 3. Data Analysis

The data gathered from YouTube videos was summed up utilizing a standard form and entered in Microsoft Excel 2016. Data analysis was performed on SPSS version 24.0. Descriptive statistics like mean and standard deviation were used to present the output of analyzed data. For the analysis of variance, statistical *t*-test was applied to find out the *t*-value and determine significant differences. Pearson's correlation coefficient (*r*) was used to find a correlation of like versus dislikes. *P* value of less than 0.05 was statistically significant at 95% confidence interval.

## 4. Results

After initial evaluation of the 109 shortlisted YouTube videos by three subject specialists, 42 (38.5%) videos were considered as relevant and were in the English language. Out of these 42 videos, 30 (71.43%) videos were excluded due to poor sound or picture quality, not presented by a faculty member, tooth preparation was not done on permanent teeth, negative comments were more than five in number, or the information about crown preparation was not adequate. Only 12 out of 109 videos (11%) were found to be educationally useful and possessed relevant information about crown preparation, and one out of these 12 videos was animated (Table 1). The links, views, likes, dislikes, and comments of all the 12 videos that met the inclusion criteria are presented in Table 2.

Total mean duration of these educationally useful videos was 971.67 s (SD = 459.66) (Table 3). The mean views/day of all the included videos in the current study were 43.55 (SD = 64.31). A mean of “likes” was 165.25 (SD = 239.01) for the 12 videos included in the current study, which showed that the average number of likes of the educationally useful “crown preparation videos” differs according to comment or not (*t* = −3.63, *P* = 0.02). The average number of “likes” for videos by the viewers/day was 1.12 (SD = 1.62). Probably because login information is required to “like,” “dislike,” or comment on any YouTube video, so most of the users just view the videos and cannot mark on “like” or “dislike” and also could not make any comments.

The mean of “dislike” of educationally useful videos was 5.92 (SD = 9.25) which also showed that not much difference of opinions was present with the comment or not comment (*t* = −0.97 and *P* = 0.35).

In Table 3, concerning the positive comments of the each educationally useful video, the mean was 2.50 (SD = 3.78), and the mean of negative comments was 0.08 (SD = 0.29) in the current study.

The correlation between the total 12 educationally useful videos, the number of views, and the number of viewers/day was positive and high, and the result was statistically significant (*r* = 0.78, *P* < 0.001). Likewise, a significant correlation was found between the total views and the like/day (*r* = 0.68, *P* = 0.02), a significant correlation was found between the total views and the positive comments/like (*r* = 0.65, *P* = 0.02), significant correlation was found with video merit (*r* = 0.75, *P* = 0.01), but no correlation was found with video scores (*r* = 0.27, *P* = 0.40) (Tables 2 and 4). The total likes of all the 12 videos and the number of views/day showed a statistically significant correlation (*r* = 0.88, *P* < 0.001), and a significant correlation was also found between the total likes and the like/day (*r* = 0.81, *P* < 0.001). However, no correlation was found between the video scores (*r* = 0.33, *P* = 0.20) and video merits (*r* = 0.37, *P* = 0.14). Also, a significant correlation was found between the total dislikes and dislike/day (*r* = 0.95, *P* < 0.001) as presented in Table 4.

## 5. Discussion

This research offers the evaluation of already available YouTube videos on the topic of crown preparation with particular focus on the quality and information in the videos and viewers' engagement, upgrading the knowledge of students to promote efficient learning.

The evolution of digital platforms for learning has led the instructors to think diversely and to upgrade the learning and teaching strategies [12]. In the current era of the pandemic, YouTube is becoming a popular information resource for the dental and healthcare community [12]. As reported by Krawczyk et al., YouTube is one of the most used sources of learning and sharing information [19].

The authors believe that this is the first report on the usefulness of YouTube videos on the topic of crown preparation. In this study, a detailed evaluation of these videos and information presented is carried out to determine their educational value. It was observed that a good number of recordings related to the crown preparation were created and uploaded anonymously with no details of the creator whether the content creator is a faculty member or not [1, 19]. Also, some videos did not follow standard procedures as given in text books and were considered unsafe to be carried out by students.

This study also observed that the viewers who liked the video also dropped a positive comment, indicating that if the information being delivered is correct, from some reliable source, and contributes to learning of the viewer, such a video will generate more comments and likes. Similar observations were reported by Ahmad and colleagues [20]. This study also suggested that educationally valuable and informative content that was delivered by specialists or doctors linked to any institutes, organizations, hospitals, clinics, or agencies was useful and helped in improving skills and knowledge of the viewers. This finding was in accordance with other studies by Ahmad et al. and Azer et al. [20, 21].

Generally, the studies show support for video-based learning as it is considered an efficient learning tool [20–23]. Moreover, it has been established in earlier studies that video-based education is an efficient way of learning [20, 23]. In addition, the students attending the lectures or practical supplemented with video learning and practicing the learnt skills on patients achieved better skills and learning strategies [20, 21].

Our research showed that the videos on YouTube that were considered useful based on likes and dislikes totaled the video score of 76% (Table 2). This means that these videos are informative and can be used as a self-learning source by the students, which is in accordance with other studies [23]. Educationally useful and academically authentic videos on YouTube are beneficial for professionals as well as students and improve the learning possibilities utilizing both audio and visual aids, and the animations further build up the interest in the subject [8, 20, 22]. According to another research, learners utilize their self-study time or time spent on the internet to learn techniques from the available interesting and clear videos [22, 24].

Our study confirms the findings of other studies [15, 22] that most of the viewers of crown preparation videos were either dental students or dental professionals and very few were from the general population. Moreover, it was observed that most of the comments dropped are to inquire about the information in the videos and not just appreciation [22]. This study also noted that majority of the viewers did watch the video but did not comment, like, or dislike as in another study [22]. The reason for this may be that to like or dislike, sign-in to the site is required, and for comment, the identity of the viewer has to be revealed [15, 22].

The merit score of crown preparation videos as devised from positive or negative comments was 38.9%. The negative remarks on the videos were mostly addressed to further improve the quality and to use accurate and relevant keywords for search of the video [22, 25], whereas the positive remarks mentioned about the quality of knowledge and information provided, clarity, and presentation skills [22, 25]. Recently, due to the pandemic, there has been a shift towards digital learning and increased use of the internet and specifically YouTube to acquire knowledge and learn skills [13, 22]. The goal of delivering knowledge online is attainable, given that all the dental professionals and specialists make efforts to provide improved quality content and information for the students [13, 22].

The authors believe that the topic of “crown preparation” is appealing only to the dental community. Hence, the YouTube videos identified and included in this study were not very high in number. Another limitation is that the content assessable on YouTube varies, and recordings are uploaded and removed constantly. Subsequently, the results may change depending upon the time period of search. In the future, the research can be done to adopt a longitudinal or field-based approach to evaluate the value of YouTube as a mode of learning for students. For further improvement in video-based distant learning, virtual simulators can be used along with video-based education to replicate the clinical condition and enhance practical skills. This will not only provide learners with a realistic situation but also give feedback to both, the instructor and students [26].

## 6. Conclusion

When used for educational purposes, YouTube can enhance the learning experience. It provides enhanced auditory and visual learning by use of eye-catching animations, videos, and pictorial explanation of content. Although the practical skills can only be learnt on simulators, extracted teeth, or on patient, but background knowledge plays a key role in accurate performance of the procedure. The viewers should be vigilant about the origin of the information being watched to avoid any misleading information. Moreover, the content creators should be more responsible when creating the videos to pass on the authentic knowledge from the educational point of view to make online learning via the YouTube platform more beneficial.

## Figures and Tables

**Figure 1:**
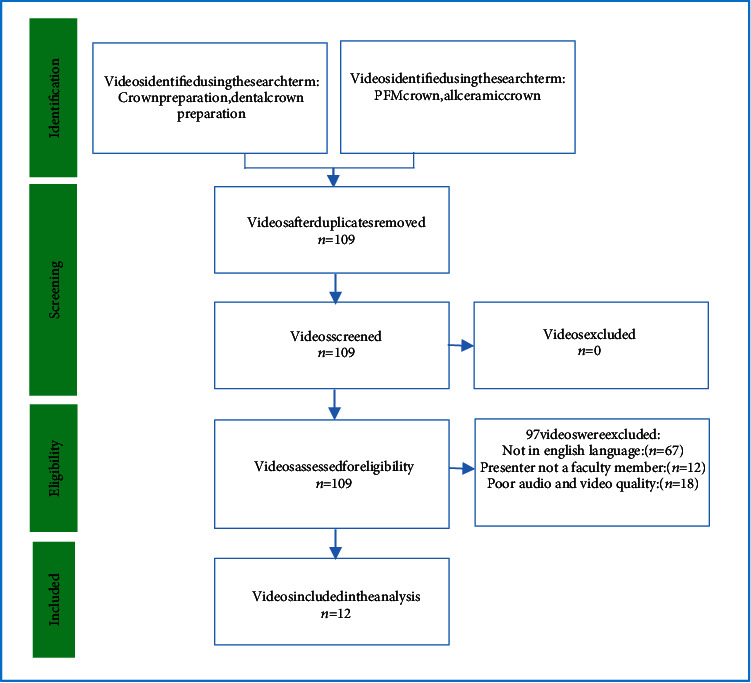
Search strategy.

**Table 1:** Detailed information of all 12 videos included in the study.

S. No.	Author (publisher/uploader)	Length (min)	SEC	Viewer/day	Like/day	Like/viewer	Dislike/day	Dislike/viewer	Positive comment/like	Negative comment/dislike
1	Glidewell Dental	15:40	940	102.97	3.16	0.03	0.08	0.00	0.02	0
2	Glidewell Dental (animated)	8:28	508	69.36	2.29	0.032	0.051	0.00	0.02	0.2
3	Glidewell Dental	8:14	494	205.16	4.94	0.02	0.20	0.00	0.02	0
4	UofU dentistry/education	22:07	1327	3.28	0.08	0.03	0	0	0	0
5	UofU dentistry/education	11:24	684	2.80	0.10	0.04	0	0	0	0
6	UofU dentistry/education	15:33	933	5.06	0.01	0.00	0.14	0.02	0	0
7	The E-Dentist	13:00	780	0.20	0.00	0.02	0	0	0	0
8	UofU dentistry/education	7:33	453	1.99	0.08	0.04	0	0	0	0
9	Stevenson Dental Solutions	23:50	1430	99.39	1.84	0.019	0.02	0.00	0.015	0
10	Dentist 4 smile	12:48	768	0.01	0	0	0	0	0	0
11	Ruiz Dental Seminars Inc.	32:09	1929	4.04	0.11	0.028	0	0	0	0
12	Stevenson Dental Solutions	23:34	1414	28.31	0.81	0.029	0.01	0.00	0.01	0

**Table 2:** Links, views, likes, dislikes, and comments of the 12 videos that met the inclusion criteria.

S. No.	Link	Total view	Like	Dislike	Positive comment	Negative comment	Video score	Video merit
1	https://www.youtube.com/watch?v=jP5d7PIk5-8	9,734	291	7	5	0	95.30	62.5
2	https://www.youtube.com/watch?v=J7LiA291R6s	6,797	224	5	5	1	95.63	26.67
3	https://www.youtube.com/watch?v=fos6C_YaRUQ	23,798	573	21	9	0	92.92	42.85
4	https://www.youtube.com/watch?v=V7dBz91xU7Q	528	13	0	0	0	100	0
5	https://www.youtube.com/watch?v=I7HPEzT3b5g	361	13	0	0	0	100	0
6	https://www.youtube.com/watch?v=7EHnV9xWzd4	870	1	26	0	0	-92.59	0
7	https://www.youtube.com/watch?v=da3eeSq4KlI	45	1	0	0	0	100	0
8	https://www.youtube.com/watch?v=qSJsVL7-9Qs	287	12	0	0	0	100	0
9	https://www.youtube.com/watch?v=Q0fJtRvq2Q4	37,074	685	9	10	0	97.40	10.52
10	https://www.youtube.com/watch?v=pKSaQG8p2Q0	3	0	0	0	0	0	0
11	https://www.youtube.com/watch?v=0gLPwHrWwcE	678	19	0	0	0	100	0
12	https://www.youtube.com/watch?v=7PBB8aeVWrA	5,295	151	1	1	0	98.68	5.55

**Table 3:** Mean sum and relevant knowledge of 12 videos that met the inclusion criteria.

Themes	Mean	(SD)	*T*	(*P*)
Total views	7100.75	(11683.18)	-2.64	(0.06)
Like	165.25	(239.01)	-3.63	(0.02)
Dislike	5.92	(9.25)	-0.97	(0.35)
Total comments	13.08	(27.01)	-1.96	(0.12)
Positive comments	2.50	(3.78)	-3.72	(0.02)
Negative comments	0.08	(0.29)	-1.00	(0.37)
Days	177.08	(78.80)	0.14	(0.89)
Video length	971.67	(459.66)	0.09	(0.93)
Viewer/day	43.55	(64.31)	-3.37	(0.03)
Like/day	1.118	(1.62)	-3.66	(0.02)
Like/viewers	0.0240	(0.01)	-0.65	(0.53)
Dislike/viewers	0.0027	(0.01)	0.72	(0.49)
Dislike/day	0.0422	(0.07)	-1.29	(0.23)
Positive comments/like	0.0064	(0.01)	-6.02	(*P* < 0.001)
Negative comments/dislike	0.0167	(0.06)	-1.00	(0.37)
Video score	76.15	(65.06)	-1.57	(0.17)
Video merit	38.89	(48.89)	-14.00	(*P* < 0.001)

*Video* *scores* = ((*like* − *dislike*)/(*like* + *dislike*))∗100. *Video* *merits* = ((*positive* *comments* − *negative* *comments*)/*total* *comments*)∗100.

**Table 4:** Correlation between the likes/dislikes, comments, video score, and merit of 12 videos.

	Viewer/day	Like/day	Dislike/day	Positive comments/like	Negative comments/dislike	Video score	Video merit
Total views	.78^∗∗^ (*P* < 0.001)	.68^∗^ (0.02)	0.38 (0.23)	.65^∗^ (0.02)	-0.01 (0.98)	0.27 (0.40)	.75^∗∗^ (0.01)
Like	.88^∗∗^ (*P* < 0.001)	.81^∗∗^ (*P* < 0.001)	0.46 (0.13)	.77^∗∗^ (0.00)	0.08 (0.81)	0.33 (0.30)	.84^∗∗^ (*P* < 0.001)
Dislike	0.57 (0.05)	0.52 (0.08)	.95^∗∗^ (*P* < 0.001)	0.32 (0.31)	-0.03 (0.92)	-0.52 (0.08)	0.32 (0.32)
Positive comments	.91^∗∗^ (*P* < 0.001)	.85^∗∗^ (*P* < 0.001)	0.50 (0.10)	.84^∗∗^ (*P* < 0.001)	0.21 (0.52)	0.32 (0.31)	.82^∗∗^ (*P* < 0.001)
Negative comments	0.13 (0.70)	0.23 (0.48)	0.04 (0.90)	.59^∗^ (0.05)	1.00^∗∗^ (*P* < 0.001)	0.14 (0.67)	0.18 (0.58)

^∗^Correlation is significant at level 5%.; ^∗∗^correlation is significant at level 1%.

## Data Availability

The data is available on request from the corresponding author.
